# The cerebellum and its network: Disrupted static and dynamic functional connectivity patterns and cognitive impairment in multiple sclerosis

**DOI:** 10.1177/1352458521999274

**Published:** 2021-03-08

**Authors:** Menno M Schoonheim, Linda Douw, Tommy AA Broeders, Anand JC Eijlers, Kim A Meijer, Jeroen JG Geurts

**Affiliations:** Department of Anatomy and Neurosciences, MS Center Amsterdam, Amsterdam Neuroscience, Amsterdam UMC, Vrije Universiteit Amsterdam, Amsterdam, The Netherlands; Department of Anatomy and Neurosciences, MS Center Amsterdam, Amsterdam Neuroscience, Amsterdam UMC, Vrije Universiteit Amsterdam, Amsterdam, The Netherlands; Department of Anatomy and Neurosciences, MS Center Amsterdam, Amsterdam Neuroscience, Amsterdam UMC, Vrije Universiteit Amsterdam, Amsterdam, The Netherlands; Department of Anatomy and Neurosciences, MS Center Amsterdam, Amsterdam Neuroscience, Amsterdam UMC, Vrije Universiteit Amsterdam, Amsterdam, The Netherlands; Department of Anatomy and Neurosciences, MS Center Amsterdam, Amsterdam Neuroscience, Amsterdam UMC, Vrije Universiteit Amsterdam, Amsterdam, The Netherlands; Department of Anatomy and Neurosciences, MS Center Amsterdam, Amsterdam Neuroscience, Amsterdam UMC, Vrije Universiteit Amsterdam, Amsterdam, The Netherlands

**Keywords:** Multiple sclerosis, cerebellum, cognition, network, connectivity, atrophy

## Abstract

**Background:**

The impact of cerebellar damage and (dys)function on cognition remains understudied in multiple sclerosis.

**Objective:**

To assess the cognitive relevance of cerebellar structural damage and functional connectivity (FC) in relapsing-remitting multiple sclerosis (RRMS) and secondary progressive multiple sclerosis (SPMS).

**Methods:**

This study included 149 patients with early RRMS, 81 late RRMS, 48 SPMS and 82 controls. Cerebellar cortical imaging included fractional anisotropy, grey matter volume and resting-state functional magnetic resonance imaging (MRI). Cerebellar FC was assessed with literature-based resting-state networks, using static connectivity (that is, conventional correlations), and dynamic connectivity (that is, fluctuations in FC strength). Measures were compared between groups and related to disability and cognition.

**Results:**

Cognitive impairment (CI) and cerebellar damage were worst in SPMS. Only SPMS showed cerebellar connectivity changes, compared to early RRMS and controls. Lower static FC was seen in fronto-parietal and default-mode networks. Higher dynamic FC was seen in dorsal and ventral attention, default-mode and deep grey matter networks. Cerebellar atrophy and higher dynamic FC together explained 32% of disability and 24% of cognitive variance. Higher dynamic FC was related to working and verbal memory and to information processing speed.

**Conclusion:**

Cerebellar damage and cerebellar connectivity changes were most prominent in SPMS and related to worse CI.

## Introduction

MS is a common neuroinflammatory and neurodegenerative disease of the central nervous system (CNS), affecting the grey matter (GM) and white matter (WM). A brain region commonly excluded in MS research is the cerebellum,^1^ especially cerebellar cortex. Previous studies showed that cerebellar cortex is commonly demyelinated^2^ and atrophic,^3–5^ with ongoing discussion on specific stagings of damage.^2,6^ In addition, recent studies on the healthy brain discovered strong cerebellar connections with specific functional networks like the fronto-parietal network (FPN)^7^ and relations with cognition.^8^ Nonetheless, in MS research, how cerebellar pathology influences cognition remains unclear.^1^

The field of network neuroscience^9^ recently evolved with the discovery of dynamic^10^ (or time-varying) functional connectivity (FC), which is strongly related to cognition.^11,12^ Conceptually, static connectivity could represent the amount of information transferred (the total correlation between two signals), while dynamic connectivity could assess the variability of the level of information transfer. As such, these two measures together could provide unique information on network functioning crucial for cognition.^13^ Unfortunately, a few studies specifically investigated the cognitive role of cerebellar FC, and even fewer in MS.^14–17^

As such, we investigated at which disease stage cerebellar cortical damage (i.e. diffusion changes and atrophy) and FC alterations (static and dynamic) become apparent in patients with relapse-onset MS and how these relate to cognition. We expected that the cerebellum would show strong disconnection in progressive MS (based on static FC), combined with highly variable cognitive connections (based on dynamic FC). This hypothesis was addressed in a large MS cohort divided into patients with a relatively short disease duration (‘early’), those with longer disease durations (‘late’) and progressive MS.

## Methods

### Participants

Retrospective data from participants of the Amsterdam MS cohort^18^ with sufficient cerebellar coverage (see functional MRI processing) and relapse-onset MS were included. Based on disease duration, the relapsing-remitting multiple sclerosis (RRMS) group was subdivided into ‘early’ (<15 years) and ‘late’ (>15 years) RRMS. The shortest disease duration was 4.6 years. The final sample (see Table 1) included 278 MS patients (74% women, age 47 ± 11 years, 149 early and 81 late RRMS, and 48 SPMS) and 82 matched healthy controls (HCs, 63% women, age 46 ± 11 years). Patients were diagnosed with the revised McDonald criteria.^19^ The Expanded Disability Status Scale (EDSS)^20^ was used to measure overall disability. Patients were relapse-free and without steroid treatment for at least 2 months prior to participating in the study. Approval was obtained from the local institutional ethics review board, and the subjects gave written informed consent prior to participation.

**Table 1.** Subject demographics, cognition and MRI measures.

	Healthy controls (*N* = 82)		Early RRMS (*N* = 149)		Late RRMS (*N* = 81)		SPMS (*N* = 48)	
	Mean	SD	Mean	SD	Mean	SD	Mean	SD
*Demographics*								
Age (years)	45.93	10.79	41.39	9.29^b^	51.97	8.54^b^	55.05	8.38^b,*^
Sex (% women)	63		73		79		69	
Education^a^	6.00	1.0–7.0	5.00	1.0–7.0	5.00	1.0–7.0	4.00	1.0–7.0
Disease duration (years)			7.93	2.60	21.78	4.65	21.26	9.46^*^
EDSS^a^			2.50	0–6	3.00	1–7.5	6.00	2.5–8.0^b,*^
*Cognition (Z-scores)*								
Executive functioning	−0.05	0.73	−0.67	1.19^b^	−1.05	2.16^b^	−1.42	1.69^b,*^
Verbal memory	0.00	0.87	−0.34	1.02	−0.41	1.20	−0.85	1.28^b^
IPS	−0.06	0.92	−0.79	1.25^b^	−1.23	1.34^b^	−1.73	1.37^b,*^
Verbal fluency	−0.04	1.00	−0.31	1.07	−0.57	0.96	−0.71	1.10
Visuospatial memory	0.00	0.91	−0.35	1.15	−0.74	1.18^b^	−0.95	1.06^b^
Working memory	0.00	0.85	−0.68	1.09^b^	−1.06	1.76^b^	−1.73	1.35^b,*^
Attention	−0.07	0.66	−0.51	0.87^b^	−0.78	1.25^b^	−0.67	1.11^b^
Average cognition	−0.03	0.48	−0.53	0.73^b^	−0.85	1.09^b^	−1.19	0.94^b,*^
*Structural damage*								
WB-LV (mL)^a^			7.05	0.59–40.77	14.75	0.93–60.08	18.79	2.06–84.85^*^
C-LV (mL)^a^			0	0–0.34	0	0–0.23	0	0–0.11
NBV (L)	1.51	0.07	1.48	0.06^b^	1.43	0.08^b^	1.41	0.08^b,*^
Cerebellar NGMV (L)	0.11	0.01	0.11	0.01	0.10	0.01^b^	0.09	0.01^b,*^
Cerebellar FA	0.19	0.02	0.18	0.02^b^	0.17	0.02^b^	0.17	0.02^b,*^

MRI: magnetic resonance imaging; SD: standard deviation; GLM: general linear model (main effect); education: highest level of education attained (on a scale of 1–7); EDSS: Expanded Disability Status Scale; IPS: information processing speed; WB-LV: T2-lesion volume of the whole brain; C-LV: T2-lesion volume in the cerebellum; NBV: normalized brain volume; NGMV: normalized grey matter volume; FA: grey matter fractional anisotropy; cerebellar connectivity: averaged static connectivity of the cerebellum with the rest of the brain.

a The values are represented in median and range.

b Indicates values significantly different from healthy control values.

* Indicates a significant difference between secondary progressive and early relapsing-remitting multiple sclerosis (both at *p* < 0.05, corrected).

### Neuropsychological evaluation

Subjects underwent neuropsychological evaluation on the day of magnetic resonance (MR) scanning using an expanded Brief Repeatable Battery of Neuropsychological (BRB-N) tests. Executive functioning (EF, concept shifting test), verbal memory (VM, selective reminding test), verbal fluency (VF, word list generation), information processing speed (IPS, symbol-digit modalities test), visuospatial memory (VSM, spatial recall test), attention (Stroop colour-word test) and working memory (WM, memory comparison test) domains were included, as described in Eijlers et al.^18^ Raw cognitive scores were corrected for normal effects of sex, age and education.^18^ *Z*-scores were calculated based on the mean values and standard deviations of the HC group for each subject. For descriptive purposes, all *Z*-scores were averaged to form an ‘averaged cognition’ score.

### Magnetic resonance imaging

**Figure 1.**
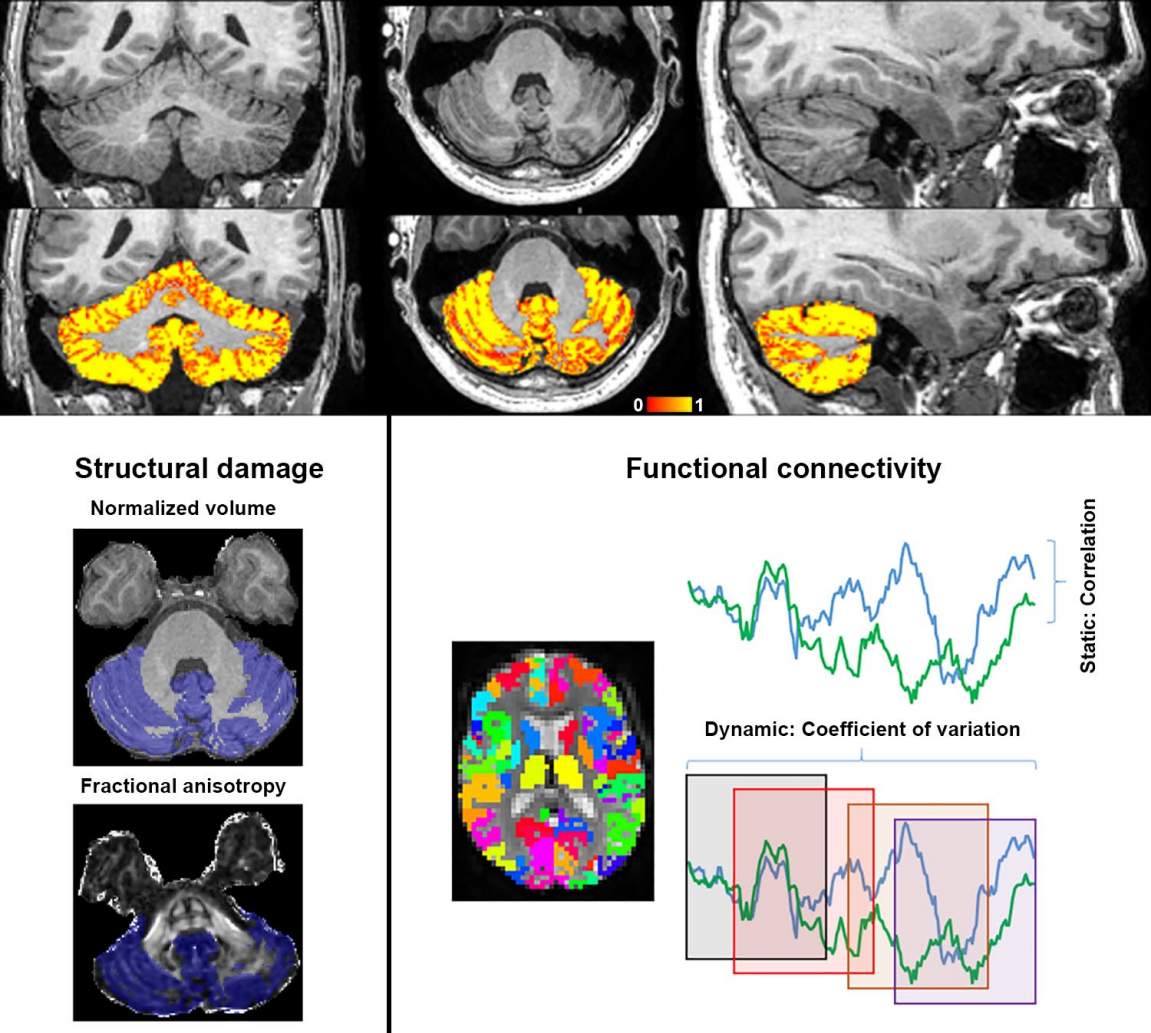
Overview of the cerebellar imaging pipeline. Top panel: cerebellar grey matter partial volume estimation in a healthy control (ranging from low (red) to high (yellow) percentage grey matter, *X* = 56, *Y* = 86 and *Z* = 39). Left panel: within the binarized mask of segmented cerebellar cortex (blue), normalized grey matter volume and mean fractional anisotropy values are calculated. Right panel: cerebellar cortical connectivity is calculated with all regions of the cortical Brainnetome and deep grey matter FIRST atlases. Static connectivity is calculated using correlation coefficients between region pairs across the entire scan. Dynamic connectivity is calculated using the coefficient of variation of connectivity values across windows (examples depicted as squares).

Subjects underwent 3T MRI (GE Signa HDxt), including three-dimensional (3D) T1-weighted fast spoiled gradient-echo (TR = 7.8 ms, TE = 3.0 ms, TI = 450 ms, FA = 12°, 0.9 mm × 0.9 mm × 1 mm voxel size) and a 3D fluid-attenuated inversion-recovery sequences (FLAIR, TR = 8000 ms, TE = 125 ms, TI = 2350 ms, 1.2 mm sagittal slices and 0.98 mm × 0.98 mm in-plane resolution). Diffusion tensor imaging based on echo planar imaging (EPI) covered the entire brain, using five volumes without directional weighting (i.e. b0) and 30 volumes with non-collinear diffusion gradients (i.e. 30 directions, *b* = 1000 s/mm^2^, TR = 13,000 ms, TE = 91 ms, FA = 90°, 53 contiguous axial slices of 2.4 mm and in-plane resolution 2 mm × 2 mm). Resting-state (i.e. eyes closed and no task) functional magnetic resonance imaging (MRI) covered the entire brain, using 202 volumes, of which the first two were discarded (EPI, TR = 2200 ms, TE = 35 ms, FA = 80°, 3 mm contiguous axial slices and in-plane resolution 3.3 mm × 3.3 mm). Figure 1 shows an overview of the processing pipeline.

### Structural MRI processing: cerebellar GM volume

As described in Eijlers et al.,^18^ WM lesions were segmented on FLAIR using the *k*-nearest neighbour classification with tissue-type priors (kNNTTP) yielding lesion maps for lesion volumes and for lesion filling on 3D-T1, to minimize their impact on processing steps. Normalized brain volumes (NBVs) were analysed using SIENAX (part of FSL5). The cerebellar region of interest (ROI) from the Harvard–Oxford atlas (part of FSL) was non-linearly co-registered to each subject’s 3D-T1 using inverted FNIRT registration parameters. To specifically investigate cerebellar GM, the binary cerebellar mask was multiplied with SIENAX’s GM partial volume estimation (PVE) image. This cerebellar GM PVE map was averaged to calculate the mean quantity of cerebellar GM, multiplied by the number of cerebellar GM voxels and normalized for head size with the V-scaling factor of SIENAX to obtain normalized cerebellar grey matter volume (GMV). The unthresholded cerebellar PVE image was binarized to form the cerebellar cortical ROI for diffusion and FC measurements (see below).

### Structural MRI processing: cerebellar cortical fractional anisotropy

Diffusion images were pre-processed using eddy current and motion correction with FSL, providing fractional anisotropy (FA) maps. Boundary-based registration (BBR) was used to calculate registration parameters between b0 images and 3D-T1 images. These were inverted and applied on the cerebellar ROI, using nearest-neighbour interpolation, to calculate mean FA within the cerebellar cortex.

### Functional MRI processing and atlas

Functional pre-processing used FSL, including basic motion correction and smoothing. Advanced motion correction was subsequently performed using ICA-AROMA, as well as WM and cerebrospinal fluid (CSF) regression and high-pass filtering (100 seconds cut-off); resting-state data were kept in subject space. Cortical regions were defined using the standard space Brainnetome atlas. Similar to the cerebellar mask, the atlas was registered to subject space using inverted non-linear registration parameters and masked with binarized SIENAX-derived GM PVE maps, before adding deep grey matter (DGM) regions derived from FIRST and the cerebellar mask. The complete GM atlas was then co-registered to the subject’s functional scan, using an inverted BBR matrix. All registration steps used nearest-neighbour interpolation. As described in Meijer et al.,^21^ for each subject, effects of EPI distortion were assessed by calculating the number of voxels within each ROI that contained reliable signal, excluding those with <30% coverage, resulting in the exclusion of bilateral orbitofrontal and nucleus accumbens areas. All subjects had at least 60% cerebellar coverage on functional magnetic resonance imaging (fMRI); mean cerebellar coverage was not significantly different between groups and was >75% in each group. For both static and dynamic connectivity measurements (see below), connections were averaged into seven well-known resting-state networks according to maximum overlap with the previous literature.^22^ These variables therefore represent static and dynamic cerebellar connectivity with the default-mode (DMN), fronto-parietal (FPN), ventral and dorsal attention (VAN and DAN), sensorimotor (SMN), visual (VN) and DGM networks. In addition, these variables were averaged to represent one global measure of static and dynamic cerebellar connectivity.

### Functional MRI processing: static and dynamic FC

For each of the remaining regions of interest in the atlas, the average signal intensity was calculated for each volume, creating 197 averaged time-series. Static FC was calculated by correlating cerebellar time-series with each of the 196 cerebral atlas regions. Negative correlations were made absolute. Whereas static FC represents the strength of the functional connection that is measured across the entire scan, dynamic FC is a measurement of variability of this functional connection strength over time.^10^ Such a measure can be interpreted as a measure of stability (i.e. a low variability) or flexibility (i.e. a high variability) of functional communication, although it should be noted that it remains unclear whether a high or low time-varying FC is to be considered ‘optimal’, and that this variability in fact seems to be network and state-dependent.^11^ To calculate dynamic FC, an in-house MATLAB script was used,^11^ which uses a sliding window approach in order to calculate FC values in a range of partially overlapping windows for each of the investigated cerebellar functional connections. Subsequently, the coefficient of variation of these connectivity values was calculated across time windows, by dividing the standard deviation across windows by the average FC, and used as a normalized measure of dynamics. Similar to previous studies, a window length of 60 seconds and a shift of 9 seconds were used.^11^

FC variables were compared with null-model data to assess whether the observed dynamics were statistically different from random noise. These models were created using phase-randomization of our data,^23^ and FC was averaged over 50 randomization runs. Randomized variables were compared to the empirical data using paired *t*-tests.

### Statistical analyses

All statistical analyses were performed in SPSS 22. Normality was assessed using the Kolmogorov–Smirnov testing and histogram inspection. Since linear models ideally incorporate variables with normal distributions, some variables needed mathematical transformation to achieve normality (static FC with log_10_(*x*), due to a right-tailed distribution, and dynamic FC with *x*^2^, due to a left-tailed distribution, see Supplementary Figure). Multivariate general linear models (GLMs) compared imaging measures between groups, including age, sex and level of education as covariates. Significant cerebellar measures were subsequently related to EDSS and cognition using two multivariate linear regression models with backward selection. Individual cognitive domains were related to FC variables that were significant in the cognition model only, using Pearson’s correlations. All reported *p*-values are Bonferroni-corrected for multiple comparisons.

## Results

### Demographics, cognition and disability

Table 1 shows all individual variables and group statistics. Early RRMS patients were younger than controls, and performed worse on all cognitive domains (average cognition *Z* = −0.53) except VM, VF and VSM. Late RRMS patients were older than controls and had deficits in all cognitive domains (average cognition *Z* = −0.85) except VM. SPMS patients were older than controls and affected on all cognitive domains (average cognition *Z* = 1.19), except VF. Disability was worst in SPMS (median EDSS = 6.0), and mild in early (2.5) and late RRMS (3.0).

### Structural cerebellar measures

Cerebellar cortical atrophy was only seen in late RRMS (−9%, *p* < 0.001) and SPMS (12%, *p* < 0.001), compared to controls. Cerebellar cortical FA was lower in early RRMS (−2%, *p* = 0.047), late RRMS (−6%, *p* = 0.006) and SPMS (−9%, *p* < 0.001), compared to controls. Cerebellar GMV related to worse EDSS (*r* = −0.39, *p* < 0.001) and all cognitive domains (average cognition *r* = 0.39, *p* < 0.001), except VF, with strongest correlations for IPS (*r* = 0.36, *p* < 0.001) and WM (*r* = 0.33, *p* < 0.001). Cerebellar cortical FA related to EDSS (*r* = −0.27, *p* < 0.001) and cognition (average cognition *r* = 0.24, *p* < 0.001), especially WM (*r* = 0.21, *p* = 0.01), IPS (*r* = 0.21, *p* = 0.01) and VSM (*r* = 0.19, *p* = 0.04), with a trend for attention (*r* = 0.18, *p* = 0.06) but no effect for EF and VF. Cerebellar WM lesion volumes were not different between groups.

### Static cerebellar connectivity: the ‘strength’ of connections

**Figure 2.**
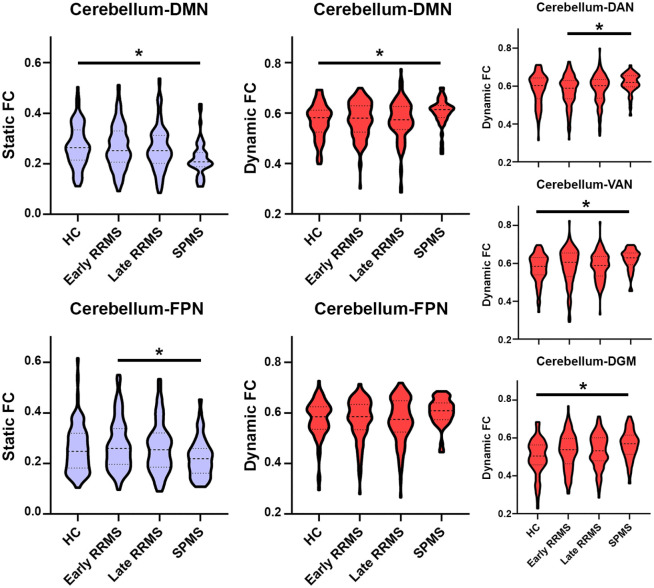
Cerebellar connectivity changes with resting-state networks in MS. HC: healthy controls; RRMS: relapsing-remitting multiple sclerosis; SPMS: secondary progressive multiple sclerosis; DMN: default-mode network; FPN: fronto-parietal network; DAN: dorsal attention network; VAN: ventral attention network; DGM: deep grey matter. *Indicates a significant effect (Bonferroni-corrected).

Main effects for static cerebellar connectivity were only seen in the DMN (*p* = 0.02) and FPN (*p* = 0.03). Cerebellum–DMN connections were lower in SPMS only (*p* = 0.017, see Figure 2) compared to controls; cerebellum–FPN connections were lower only in SPMS compared to early RRMS (*p* = 0.019).

### Dynamic cerebellar connectivity: the ‘variability’ of connections

Main effects for dynamic cerebellar connectivity were seen in the DMN (*p* = 0.04), DAN (*p* = 0.05), VAN (*p* = 0.03) and DGM (*p* = 0.004). These cerebellar connections showed higher dynamic FC (see Figure 2) in SPMS compared to HC (DMN, *p* = 0.04; VAN, *p* = 0.02; DGM, *p* = 0.009) and compared to early RRMS (DAN, *p* = 0.03). In addition, cerebellum–DGM dynamic FC was higher in early RRMS compared to HC (*p* = 0.03).

### Linear regression: disability and cognition

**Figure 3.**
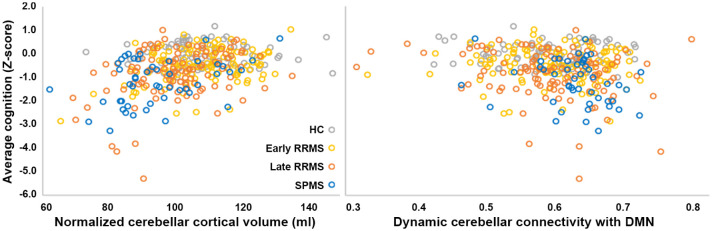
Cerebellar cortical atrophy and increased dynamic cerebellum–DMN connectivity are related to averaged cognitive performance in MS. RRMS: relapsing-remitting multiple sclerosis; SPMS: secondary progressive multiple sclerosis; DMN: default-mode network.

Backward linear regression models performed in MS included age, sex, level of education, cerebellar GMV, cerebellar cortical FA, static cerebellar FC with DMN and FPN, and dynamic cerebellar FC with DMN, DAN, VAN and DGM as predictors. Worse disability related (adjusted *R*^2^ = 0.32, *F* = 27.2, *p* < 0.001) to higher age (β = 5.1, *p* < 0.001), lower education (β = −4.3, *p* < 0.001), worse cerebellar atrophy (β = 4.0, *p* < 0.001) and higher dynamic cerebellar FC with DAN (β = 3.0, *p* = 0.003) and DGM (β = 2.7, *p* = 0.008) networks. Worse cognition related (adjusted *R*^2^ = 0.24, *F* = 30.3, *p* < 0.001, see Figure 3) to worse cerebellar atrophy (β = 6.6, *p* < 0.001), lower education (β = 5.3, *p* < 0.001) and higher cerebellum–DMN dynamic FC (β = −2.6, *p* = 0.009). Static FC was not retained in either model. Higher cerebellum–DMN dynamic FC related to poorer WM (*r* = −0.19, *p* = 0.014), VM (*r* = −0.17, *p* = 0.026) and IPS (*r* = −0.16, *p* = 0.007), even after correcting for age, sex and level of education (and multiple testing). Disease duration was not related to FC, but did relate to average cognition (*r* = −0.24, *p* < 0.001), EDSS (*r* = 0.44, *p* < 0.001), as well as cerebellar cortical FA (*r* = −0.25, *p* < 0.001) and volume (*r* = −0.40, *p* < 0.001). Cerebellar lesion volume was not related to cognition, EDSS or FC, but did relate to cerebellar atrophy (rho = −0.13, *p* = 0.031) and a trend for cerebellar cortical FA (rho = −0.12, *p* = 0.053).

### Null models

Randomized data were generated for all FC variables and compared to the empirical data. While all static FC measures remained unaffected, all dynamic FC measures were significantly different from their randomized counterparts (all *p* < 0.001).

## Discussion

In this study, we investigated at which disease stage cerebellar alterations, that is, group differences in FA, GM volume and FC, become apparent in relapse-onset multiple sclerosis and how these explain cognitive impairment (CI) and disability. Cerebellar damage was mild in early RRMS, while late RRMS showed signs of cerebellar atrophy, which further worsened in SPMS. Static FC (i.e. strength of connectivity) was only lower in SPMS, while dynamic FC (i.e. variability of connectivity) was only higher in SPMS, compared to early RRMS and controls. Cerebellar dynamic FC (but not static FC) and cerebellar atrophy together explained 32% of disability (together with age and education) and 24% of cognitive variance (together with education). Dynamic connectivity correlated with disability, WM and VM and IPS.

Lower static FC of the cerebellar cortex was only seen in SPMS and only with the DMN and FPN. Both networks are known to be structurally connected with the cerebellum.^7^ Interestingly, no early connectivity changes were found. This could contradict the hypothesized construct of functional reorganization,^14^ where functional activation and connectivity are thought to increase to compensate for structural damage. Instead, these findings support the hypothesis of a network collapse in progressive MS.^24^ The few studies that assessed cerebellar function mostly showed severe cerebellar connectivity alterations in SPMS only.^16,25^ In this study, in late RRMS, cerebellar FC was still normal, while cerebellar cortical volume and FA were already lower compared to controls. These structural differences were possibly driven by demyelination and a loss of tissue organization due to loss of Purkinje cells.^3^ While some previous studies also showed cerebellar atrophy^2^ and WM integrity loss^26^ in RRMS, effects were shown to be especially evident in SPMS.^4^ Together, these findings indicate that structural cerebellar damage becomes apparent before functional cerebellar network alterations, which seem to develop in the transitional period towards SPMS. This finding could support the notion of a ‘tipping’ point in the severity of structural cerebellar damage in the later stages of RRMS, after which the network will destabilize. This sudden ‘network collapse’ could hereby explain the sudden cognitive worsening in progressive MS.^27^ Future work is required, however, to further study these effects over time, in order to pinpoint how brain function is able to remain normal in earlier stages of the disease and the specific underpinnings of this altered FC.

Dynamic FC was markedly altered in many more networks compared to effects of static FC, and again almost exclusively in SPMS. Strongest relations with cognition were found for dynamic cerebellum–DMN FC. Previous work showed that in SPMS, atrophy is prominent in regions that are known DMN hub areas, which is much worse than in RRMS^28^ and might explain this DMN specificity. We only observed higher dynamic FC, which might indicate that the connection between the cerebellum and DMN may not be sufficiently stable to properly process information, a process similar to that hypothesized to occur in brain damaged patients with a lowered level of consciousness.^29^ This increase could be an attempt to preserve normal functioning, that is, that the cerebellum and DMN continuously attempt to reconnect to preserve normal processing. However, it could also merely be the result of some form of disinhibition and not any form of active reorganization. However, our measure of dynamic FC was limited to the coefficient of variation over time of a specific connection. As such, more in-depth dynamic FC analyses could provide additional information, for instance, investigating specific cerebellar connectivity patterns and how these are organized in time. These so-called functional ‘metastates’ have previously been indicated to be important for cognition in MS, but have not been explored in the cerebellum.^30^

The specificity of altered static cerebellar connectivity with the DMN and FPN could also be explained by results of previous tract-tracing studies, showing strong structural connections between the cerebellum and these brain areas.^31^ Correlations with WM specifically, as seen in this study, seem valid, given that the FPN is directly involved in WM,^32^ as is the cerebellum itself.^33^ Interestingly, cerebellar connectivity was also related to reduced IPS in this study as well as other recent MS works,^34,35^ although the cerebellum is not traditionally implicated in this cognitive domain in studies on the healthy brain.^8^ This could represent a ‘bleed-through’ effect of WM on the symbol-digit modalities test, and/or the other way around, as well as a possible role for the cerebellum in fine-tuning cognition by affecting aspects of IPS.

Some limitations should be acknowledged. First, very early MS could not be investigated, where data remain rare. In addition, due to sample size, we could not group controls into age bins, although we included age as a covariate. Furthermore, we used one cerebellar mask, while segmenting cerebellar lobules could provide additional information. For instance, it is known that cortico-cerebellar connections are extensively mediated by the thalamus,^31^ and a previous study found reduced thalamic connectivity with specific parts of the cerebellum in MS.^15^ Specifically investigating the integrity of the cortico-thalamo-cerebellar circuit in MS might also increase statistical contrast. Finally, longitudinal assessments could investigate individual trajectories of progression, including assessments of abnormalities in specific network states.^11^

In conclusion, these novel findings indicate that cortico-cerebellar FC is especially affected in SPMS, focused on the default-mode and DGM networks. Lower static FC is accompanied by higher variability in FC strength, the latter of which especially relates to the cognitive and physical decline in this phase of the disease. These findings indicate the importance of including the cerebellum in studies investigating cognitive dysfunction, while future longitudinal studies are now required to further investigate the prognostic value and cause of these findings.

## Supplemental Material

sj-pdf-1-msj-10.1177_1352458521999274 – Supplemental material for The cerebellum and its network: Disrupted static and dynamic functional connectivity patterns and cognitive impairment in multiple sclerosis Click here for additional data file. Supplemental material, sj-pdf-1-msj-10.1177_1352458521999274 for The cerebellum and its network: Disrupted static and dynamic functional connectivity patterns and cognitive impairment in multiple sclerosis by Menno M Schoonheim, Linda Douw, Tommy AA Broeders, Anand JC Eijlers, Kim A Meijer and Jeroen JG Geurts in Multiple Sclerosis Journal
