# Supplementary material for: The cerebellum and its network: Disrupted static and dynamic functional connectivity patterns and cognitive impairment in multiple sclerosis
Source: Mult Scler. 2021 Mar 8;27(13):2031–9. doi: 10.1177/1352458521999274 (PMC8564243; doi:10.1177/1352458521999274)
Supplement: sj-pdf-1-msj-10.1177_1352458521999274 – Supplemental material for The cerebellum and its network: Disrupted static and dynamic functional connectivity patterns and cognitive impairment in multiple sclerosis [file sj-pdf-1-msj-10.1177_1352458521999274.pdf]

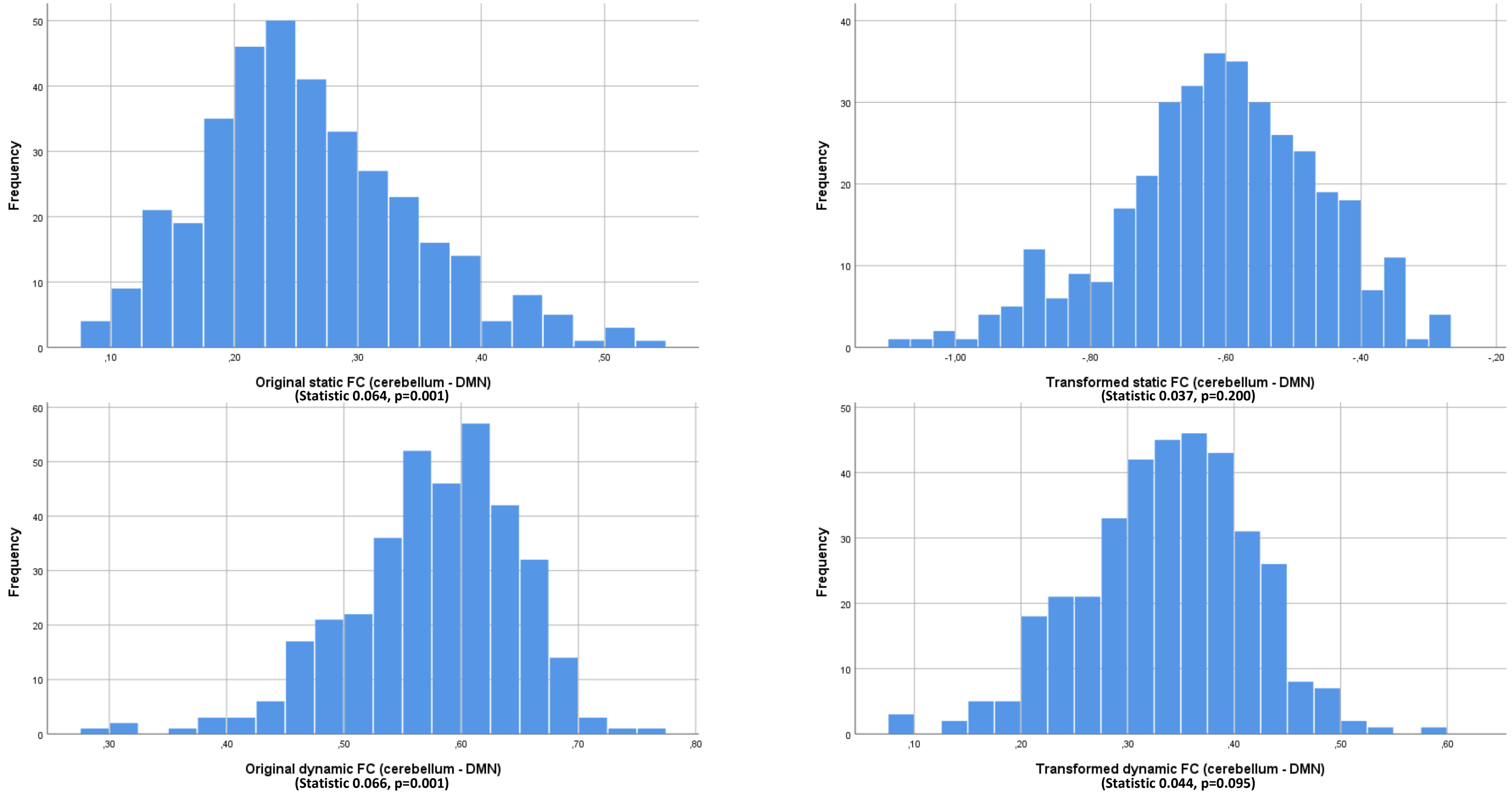

Supplementary Figure: Histograms of static and dynamic functional connectivity (FC) before (left) and after (right) transformations to ensure normal distributions. For illustrative purposes only cerebellar connections with the default-mode network (DMN) are shown. Static connectivity was transformed using log10(x), due to the observed right-tailed distribution. Dynamic connectivity was transformed using x^2, due to the observed left-tailed distribution. Statistics represent the Kolmogorov-Smirnov test for normal distributions; significance indicates a non-normal distribution.
